# Idiopathic brain calcification in a patient with hereditary hemochromatosis

**DOI:** 10.1186/s12883-020-01689-1

**Published:** 2020-03-30

**Authors:** Stefania Scarlini, Francesco Cavallieri, Massimo Fiorini, Elisa Menozzi, Francesca Ferrara, Francesca Cavalleri, Chiara Reale, Barbara Garavaglia, Antonello Pietrangelo, Franco Valzania, Elena Corradini

**Affiliations:** 1 Internal Medicine Unit and Centre for Hemochromatosis and Heredometabolic Liver Diseases, EuroBloodNet Referral Center for Iron Disorders, Policlinico, Azienda Ospedaliero-Universitaria di Modena, Modena, Italy; 2 Neurology Unit, Neuromotor & Rehabilitation Department, Azienda USL-IRCCS di Reggio Emilia, Viale Risorgimento 80, 42123 Reggio Emilia, Italy; 3 Clinical and Experimental Medicine PhD Program, University of Modena and Reggio Emilia, Modena, Italy; 4 Department of Neuroscience, S. Agostino-Estense Hospital, Azienda Ospedaliero-Universitaria di Modena, Modena, Italy; 5 Department of Neuroradiology, Policlinico|, Azienda Ospedaliero Universitaria di Modena, Modena, Italy; 6 Medical Genetics and Neurogenetics Unit, Movement Disorders Diagnostic Section, Fondazione IRCCS Istituto Neurologico “C. Besta”, Milan, Italy; 7 Department of Medical and Surgical Sciences, University of Modena and Reggio Emilia, Modena, Italy

**Keywords:** Basal ganglia, Brain calcification, Calcium, Hereditary hemochromatosis, Iron

## Abstract

**Background:**

Detection of brain-MRI T2/T2* gradient echo images (T2*GRE)-hypointensity can be compatible with iron accumulation and leads to a differential diagnosis work-up including neurodegeneration with brain iron accumulation (NBIA) and Wilson Disease. Idiopathic or secondary brain calcification can be also associated with neurological involvement and brain-MRI T2/T2*GRE-hypointensity. Hereditary hemochromatosis (HH), characterized by systemic iron loading, usually does not involve the CNS, and only sporadic cases of neurological abnormalities or brain-MRI T2/T2*GRE-hypointensity have been reported.

**Case presentation:**

A 59-year-old man came to our observation after a diagnosis of HH carried out in another hospital 2 years before. First-level genetic test had revealed a homozygous HFE p.Cys282Tyr (C282Y) mutation compatible with the diagnosis of HFE-related HH, thus phlebotomy treatment was started. The patient had a history of metabolic syndrome, type-2 diabetes, autoimmune thyroiditis and severe chondrocalcinosis. Brain-MRI showed the presence of bilateral T2*GRE hypointensities within globus pallidus, substantia nigra, dentate nucleus and left pulvinar that were considered expression of cerebral siderosis. No neurological symptoms or family history of neurological disease were reported. Neurological examination revealed only mild right-sided hypokinetic-rigid syndrome. Vitamin D–PTH axis, measurements of serum ceruloplasmin and copper, and urinary copper were within the normal range. A brain computed tomography (CT) was performed to better characterize the suspected and unexplained brain iron accumulation. On the CT images, the hypointense regions in the brain MRI were hyperdense. DNA sequence analysis of genes associated with primary familial brain calcification and NBIA was negative.

**Conclusions:**

This report highlights the importance of brain CT-scan in ambiguous cases of suspected cerebral siderosis, and suggests that HH patients with a severe phenotype, and likely associated with chondrocalcinosis, may display also brain calcifications. Further studies are needed to confirm this hypothesis. So far, we can speculate that iron and calcium homeostasis could be reciprocally connected within the basal ganglia.

## Background

Detection of brain MRI T2/T2* gradient echo images (T2*GRE) hypointensity has an extensive differential diagnosis. The most common causes of these findings are age-related, secondary or idiopathic brain calcifications. The secondary form may be due to endocrine disease with alteration of calcium and phosphate homeostasis (e.g. hypoparathyroidism, pseudo-hypoparathyroidism, hyperparathyroidism), rare brain infections (including tuberculosis and congenital toxoplasmosis), toxin exposure (such as lead and carbon monoxide), neurodegenerative changes (diffuse neurofibrillary tangles with calcification, multiple system atrophy), leukemia, trauma, autoimmune or mitochondrial disease, while the idiopathic form consists of primary familial brain calcification [1, 2]. However, T2/T2*GRE hypointensity can be also compatible with iron accumulation and leads to a differential diagnosis work-up including neurodegeneration with brain iron accumulation (NBIA) and Wilson disease. All these conditions may present with neurological involvement [3].

Hereditary hemochromatosis (HH), a syndrome characterized by systemic iron loading, usually does not involve the central nervous system (CNS), and only sporadic cases of neurological abnormalities or brain-MRI T2/T2*GRE hypointensity have been reported [4]. On the contrary, arthropathy is a common finding in HH patients, and calcification within the cartilage (chondrocalcinosis) can be observed in articular and non-articular sites [5].

## Case presentation

**Fig. 1:**
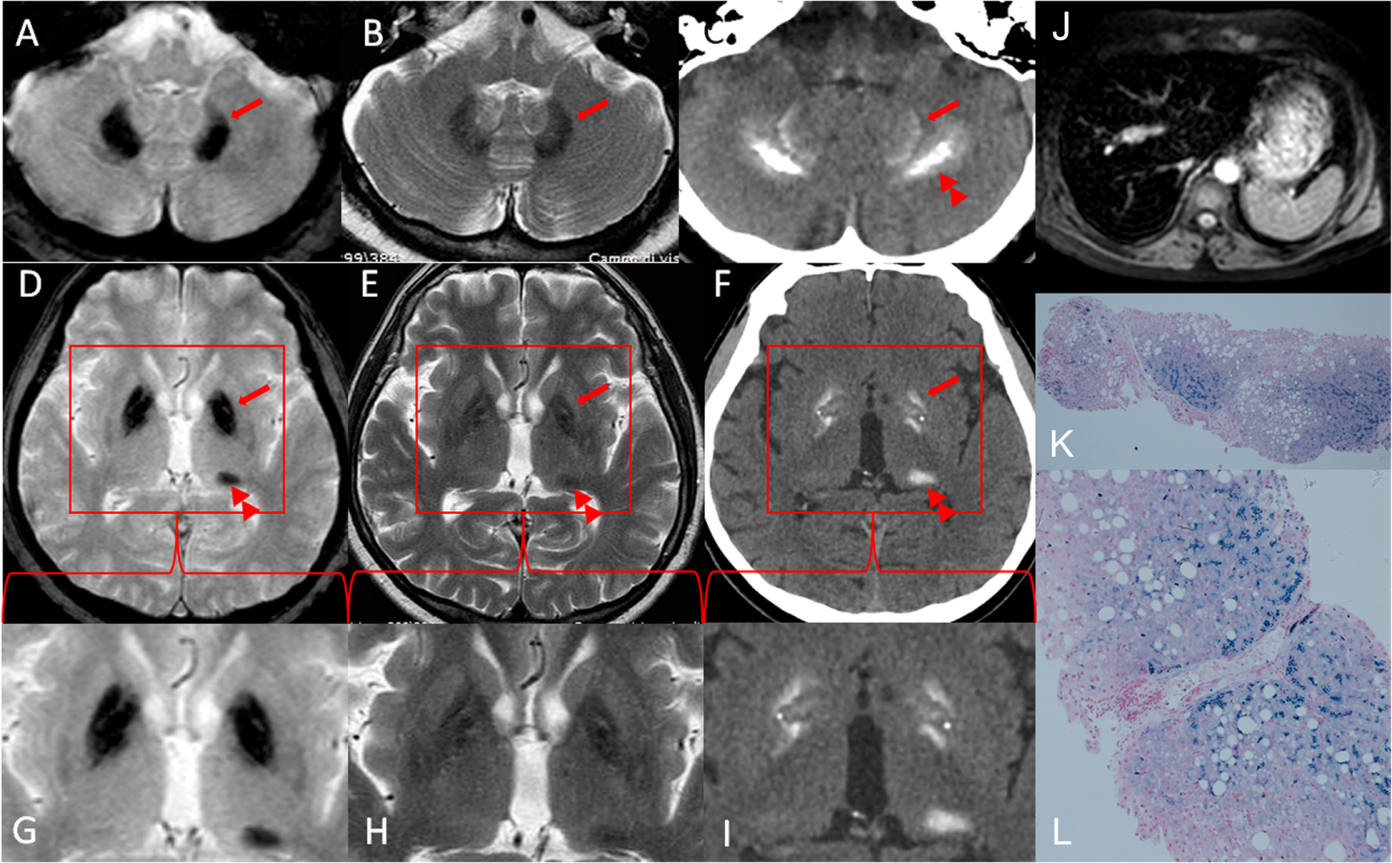
Brain-MRI, CT scan, liver biopsy and abdominal-MRI findings. **a, b** 1.5 T brain-MRI T2* gradient echo (T2* GRE) and T2-weigthed images show the presence of bilateral hypointensities within dentate nucleus (arrows) with no involvement of the peridentate white matter. **c** CT scan shows the presence of a hyperdense peripheral rim within the dentate nuclei (double arrowhead) associated with symmetrical bilateral calcification in the peridentate white matter. **d, e** 1.5 T brain-MRI T2* GRE and T2-weigthed images show the presence of bilateral hypointensities within the globus pallidus (arrow) and left pulvinar (double arrowhead). The hypointensities were more prominent in the T2* GRE sequence due to the “blooming” that results from magnetic field inhomogeneity. **f** CT scan shows the presence of a slightly hyperdense peripheral rim within the external globus pallidus (arrow) and the medial part of the internal globus pallidus (GPi) with a relative sparing of the central portion of the GPi, and left pulvinar (double arrowhead). **g-i** Magnification of the rectangular areas highlighted in images D-F. **j** T2*-weighted MRI abdominal scans show marked liver hypointensity whereas bone marrow and spleen signal intensity appears normal showing the typical MRI pattern of hereditary hemochromatosis. **k, l** Liver histopathological evaluation by Perls’ stain shows the typical parenchymal iron overload pattern of hereditary hemochromatosis: hepatocellular siderosis distributed throughout the lobule with a decreasing periportal-to-centrilobular gradient. A moderate macrovesicular steatosis and moderate periportal fibrosis are also appreciable

A 59-year-old man came to our observation for clinical and therapeutic evaluation after a diagnosis of HH carried out in another hospital 2 years before. Ferritin was reported close to 7000 ng/ml at that time. First-level genetic test had revealed a homozygous HFE p.Cys282Tyr (C282Y) mutation compatible with the diagnosis of HFE-related HH, thus phlebotomy treatment was started. The patient had a history of metabolic syndrome, type-2 diabetes on metformin treatment, autoimmune hypothyroidism on levothyroxine therapy and severe chondrocalcinosis. A previous brain-MRI performed for recurrent headache had shown the presence of bilateral T2*GRE hypointensities within globus pallidus (GP), substantia nigra, dentate nucleus and left pulvinar (Fig. 1a, b, d, e, g, h) that were considered expression of cerebral siderosis. No neurological symptoms or family history of neurological disease were reported. Blood tests performed at our Centre showed normal complete blood count (CBC) results except for a slightly reduced platelet count (144,000/mm3, normal range (n.r.) 150–450,000/mm3); biochemical evaluation confirmed altered serum iron parameters (ferritin 4728 ng/ml, n.r. 25–300 ng/ml; serum iron 282 μg/dl n.r. 50–150 μg/dl; transferrin 223 mg/dl, n.r. 200–360 mg/dl; and transferrin saturation 90%); aminotransferases were slightly elevated (AST 55 U/L, ALT 103 U/L; n.r. 1–31 U/l), with normal serum bilirubin, alkaline phosphatase, gamma glutamyl transferase; inflammatory markers and biochemical parameters of active haemolysis (LDH, haptoglobin and bilirubin) were negative; renal function and vitamin D-parathyroid hormone (PTH) axis were normal, while hypothyroidism was well compensated by hormone replacement therapy; serological screening for HAV, HBV and HCV was negative, and serum alfa1- antitrypsin level was normal. Further investigations showed: ceruloplasmin 21 mg/dl (n.r. 20–60 mg/dl) with normal plasmatic and 24-h urine copper, absence of Keyser-Fleischer ring and pigmentary retinopathy at ophthalmologic examination, and normal peripheral blood smear. Physical examination revealed an overweight Caucasian male (BMI, body mass index: 27) with mild hepatomegaly. Abdominal ultrasound and transient elastography (Fibroscan®) revealed diffuse liver steatosis and severe fibrosis (Metavir F4). Liver MRI showed severe hepatic siderosis, while biopsy confirmed the presence of severe hepatocellular iron accumulation and advanced fibrosis associated with features of non-alcoholic steatohepatitis (Fig. 1j, k, l). Heart MRI documented a mild iron overload. Based on the severity of clinical phenotype, the frequency of venesections was intensified, and analysis of other HH-associated genes (TFR2, SLC40A1, HJV and HAMP) was performed with negative results. Neurological examination revealed only mild right-sided hypokinetic-rigid syndrome. A brain computed tomography (CT) was performed to better characterize the suspected and unexplained brain iron accumulation. In the CT images, the hypointensity areas emerged at brain-MRI were partly hyperdense and compatible with bilateral brain calcifications (Fig. 1c, f, i). It is worth noting that even if the MRI hypointensity and CT hyperdensity involved the same brain areas, there was a slightly different pattern of signal distribution particularly in cerebellar and pallidal regions. In more detail, the dentate nuclei presented a bilateral hypointensity on T2*GRE and T2-weigthed images with only a slightly hyperdense peripheral rim on the CT scan, possibly related to “blooming” of iron deposits. On the contrary, the peridentate white matter is characterized by the absence of T2 or T2*GRE hypointensity but the presence of symmetrical bilateral hyperdensity compatible with calcification. The GP was bilaterally characterized by a central hypointensity with blooming effect on T2*GRE images, possibly related to iron accumulation. Instead, the CT scan showed an hyperdense peripheral rim within the lateral part of the external GP and the medial part of the internal GP with a relative sparing of the central portion of the GP. In dentate nuclei as well as in GP and pulvinar, hypointensity was more evident in T2*GRE images than in T2 images. Infectious, traumatic, or mitochondrial diseases, and hypoparathyroidism or pseudo-hypoparathyroidism were excluded on the basis of patient’s medical history and full clinical/biochemical examination, including endocrine tests. Also, the sequencing of primary familial brain calcification-associated genes (SCL20A2, PDGFB, PDGFRB, XPR1, MYORG) and NBIA genes (PANK2, PLA2G6, WDR45, FTL, C19ORF12, FA2H, COASY, CP, DCAF17, ATP13A2, FTH1, MECR, GLB1, PPCS, PPCDC, REPS1, ATP7B) was negative.

## Discussion

HH is defined as a systemic iron overload of genetic origin due to lack of synthesis or activity of hepcidin, the hormone that regulates the iron entry into the bloodstream, leading to excess circulating iron, tissue iron deposition and organ disease affecting liver, heart, endocrine glands, joints and skin. Although mutations in five different genes have been linked to the development of HH, most cases are due to the homozygous C282Y amino acid substitution in the HFE protein [6]. The main hepatic manifestations are hepatomegaly and progressive liver disease, characterized by fibrogenesis and cancer risk. Moreover, in patients with early and massive iron overload, iron-induced toxicity can lead to cardiac contractile disfunction (first diastolic and then also systolic) and electrical disturbances (mainly bradyarrhythmia, heart block or atrial fibrillation). HH related endocrinopathies include diabetes mellitus and, more rarely, hypopituitarism, adrenal insufficiency and hypothyroidism, whereas melanoderma (darkening of the skin) is the leading dermatological sign. Joint pain is frequent in HH patients and is often due to chondrocalcinosis or mono/oligo/polyarthritis, typically in the second and third metacarpophalangeal joints and ankles [6]. Usually HH does not involve the central nervous system (CNS), and only sporadic descriptions (i.e. case reports and small case series) of neurological abnormalities have been reported [4, 7, 8]. Different presentations include both neuroimaging findings suggestive of iron deposition in the absence of neurological manifestations, and neurological symptoms/signs in patients with normal MRI scan. Furthermore, a few cases of basal ganglia calcifications have been described [4, 8]. Interestingly, variable or absent responses to iron depletion are reported in these patients. The basal ganglia, in particular the putamen and GP, may represent a preferential niche for this homeostatic derangement based on their documented tendency to iron deposition and their major susceptibility to metabolic alterations due to rich vascular supply and high metabolic activity with increased utilization of glucose and oxygen [9].

Neuroimaging plays a crucial role in distinguishing NBIA (particularly PKAN) from syndrome of brain calcification. Importantly, since brain calcifications appear in MRI with various signal intensities, including T2*GRE-hypointensity that is also a typical feature of brain iron accumulation, CT-scan is considered the gold standard in their detection [3, 10]. In the present case, a missed diagnosis of cerebral calcifications was avoided through the use of CT of the brain. Notably, the prevalence of calcifications and their relationship to T2 or T2*GRE hypointensity in brain accumulation disorders is not well understood because of an underrepresentation of CT data in literature. Moreover, MRI is often the first and only imaging study for assessment of either iron accumulation or movement disorders in clinical practice.

Based on the negative results of genetic and laboratory tests, the aetiology of brain calcifications remains elusive in our patient. However, considering the severe HH phenotype (including massive hepatic iron deposition, advanced liver disease, mild heart iron overload, multiple endocrinopathies likely promoted or enhanced by iron toxicity, and chondrocalcinosis), it could be hypothesized that a local alteration of phosphate and calcium metabolism related to HH has led to calcium deposition in the basal ganglia. This hypothesis is based on previous in vitro studies where McCarty and colleagues demonstrate in red cells and synovial fluid that iron, as Fe2+ ion, inhibits pyrophosphatase, leading to a diminished hydrolysation of inorganic pyrophosphate with a consequent precipitation of inorganic pyrophosphate with calcium [11]. This mechanism, which is probably the pathophysiological process underlying chondrocalcinosis in HH, could also be a possible cause of the brain calcifications described in the present case. To further support our hypothesis, the slight morphological discrepancy between MRI and CT signal patterns in cerebellar and pallidal regions could suggest that the MRI hypointensity may be related to accumulation of both iron and calcium. Interestingly, CT hyperdense lesions within basal ganglia have been previously reported in HH patients, [4, 7, 8, 12] while more recently it has been described that patients with NBIA syndromes may present basal ganglia calcifications, [10] suggesting a close link between iron and calcium metabolism within the CNS. In our case, the calcification pattern is greater than and different from the calcification patterns seen in physiologic calcifications and in the few published cases of calcifications with HH, which appear more similar to physiologic calcifications [7, 12]. Furthermore, the additional areas of calcification appear to correspond to previously described areas of brain signal abnormality in HH summarized in Kumar et al. [4]

In addition to the standard T2*GRE sequence, susceptibility weighted imaging could provide a more sensitive evaluation of T2*GRE hypointensity and the “blooming” that results from magnetic field inhomogeneity, including from paramagnetic materials. Also, this imaging technique has some ability to differentiate different T2*GRE hypointense calcium from iron because of their different paramagnetic properties [13–15]. Unfortunately, susceptibility weighted imaging was not available in our case thus limiting the differentiation of iron and calcium brain deposits.

An understandable lack of pathology confirmation of iron and calcium is a known minor limitation in terms of interpreting findings. While not pathologically proven, the CT hyperattenuation in this case is most consistent with calcification, as previously reported in common clinical practice and in the literature [3, 10]. It is difficult to find CT-pathologic correlation in the literature for HH and brain iron accumulation, which may in part be because some or most brain iron accumulation do not demonstrate calcification [12, 16].

In conclusion this report highlights the importance of brain CT-scan in ambiguous cases of suspected cerebral siderosis, and suggests that HH patients with severe full-blown phenotype, likely associated with chondrocalcinosis, may display also brain calcifications. More work is needed to characterize the prevalence of brain calcifications in HH and how it may be related to disease diagnosis, staging, prognosis and management. So far, we can speculate that iron and calcium homeostasis could be reciprocally connected within the basal ganglia and/or cerebellum. Further studies are needed to confirm and clarify our hypothesis. On this basis, although CNS involvement is rarely described in HH patients, in presence of neurological symptoms and / or brain- MRI T2*GRE-hypointensities we recommend to always consider a CT scan not only to clarify the nature of brain deposits but also to verify if both iron and calcium are present.

## Data Availability

Data sharing is not applicable to this article as no datasets were generated or analysed during the current study.

## Abbreviations

ALT: Alanine aminotransferase
AST: Aspartate aminotransferase
ATP13A2: ATPase cation transporting 13A2
ATP7B: ATPase copper transporting beta
BMI: Body mass index
CBC: Complete blood count
C19ORF12: Chromosome 19 open reading frame 12
COASY: Coenzyme A synthase
CP: Ceruloplasmin
CT: Computed tomography
DCAF17: DDB1 and CUL4 associated factor 17
FA2H: Fatty acid 2-hydroxylase
FTH1: Ferritin heavy chain 1
FTL: Ferritin light chain
GLB1: Galactosidase beta 1
GP: Globus Pallidus
HAMP: Hepcidin gene
HAV: Hepatitis A virus
HBV: Hepatitis B virus
HCV: Hepatitis C virus
HFE: Hemochromatosis gene
HH: Hereditary haemochromatosis
HJV: Hemojuvelin gene
LDH: Lactate dehydrogenase
MECR: Mitochondrial trans-2-enoyl-CoA reductase
MRI: Magnetic resonance imaging
MYORG: Myogenesis-regulating glycosidase
NBIA: Neurodegeneration with brain iron accumulation
PANK2: Pantothenate kinase 2
PDGFB: Platelet-derived growth factor beta chain
PDGFRB: Platelet-derived growth factor receptor beta
PLA2G6: Phospholipase A2 group VI
PPCDC: Phosphopantothenoylcysteine decarboxylase
PPCS: Phosphopantothenoylcysteine synthetase
PTH: Parathyroid hormone
REPS1: RALBP1 associated Eps domain containing 1
SLC40A1: Solute carrier family 40 member 1
TFR2: Transferrin receptor 2
T2*GRE: T2* gradient echo images
WDR45: WD repeat domain 45
XPR1: Xenotropic and polytropic retrovirus receptor 1
